# Setting the stage for chronic health problems: cumulative childhood adversity among homeless adults with mental illness in Vancouver, British Columbia

**DOI:** 10.1186/1471-2458-14-350

**Published:** 2014-04-12

**Authors:** Michelle L Patterson, Akm Moniruzzaman, Julian M Somers

**Affiliations:** 1 Faculty of Health Sciences, Simon Fraser University, 8888 University Drive, Burnaby, British Columbia, Canada

## Abstract

**Background:**

It is well documented that childhood abuse, neglect and household dysfunction are disproportionately present in the backgrounds of homeless adults, and that these experiences adversely impact child development and a wide range of adult outcomes. However, few studies have examined the cumulative impact of adverse childhood experiences on homeless adults with mental illness. This study examines adverse events in childhood as predictors of duration of homelessness, psychiatric and substance use disorders, and physical health in a sample of homeless adults with mental illness.

**Methods:**

This study was conducted using baseline data from a randomized controlled trial in Vancouver, British Columbia for participants who completed the Adverse Childhood Experiences (ACE) scale at 18 months follow-up (n = 364). Primary outcomes included current mental disorders; substance use including type, frequency and severity; physical health; duration of homelessness; and vocational functioning.

**Results:**

In multivariable regression models, ACE total score independently predicted a range of mental health, physical health, and substance use problems, and marginally predicted duration of homelessness.

**Conclusions:**

Adverse childhood experiences are overrepresented among homeless adults with complex comorbidities and chronic homelessness. Our findings are consistent with a growing body of literature indicating that childhood traumas are potent risk factors for a number of adult health and psychiatric problems, particularly substance use problems. Results are discussed in the context of cumulative adversity and self-trauma theory.

**Trials registration:**

This trial has been registered with the International Standard Randomized Control Trial Number Register and assigned ISRCTN42520374.

## Background

Research into the causes of homelessness suggests complex interactions between structural and individual factors, both of which are often present long before the onset of first homelessness [1,2]. The childhoods of homeless adults are disproportionately characterized by persistent poverty, residential mobility, school problems, and other stressful and/or traumatic experiences [3-5] particularly among homeless individuals with mental illness [2]. In fact, the childhoods of homeless people with mental illness have been described as a “double dose” of disadvantage in the form of poverty as well as violence and family instability [2].

A large body of evidence suggests that adverse childhood experiences, which typically include physical, sexual, and emotional abuse, neglect, dysfunctional family environments, and unstable family structure, are linked to later psychological functioning and may affect multiple domains of health and well-being [6-8]. Moreover, it appears that adverse childhood experiences tend to cluster together [9,10] and the *number* of adverse experiences may be more predictive of negative adult outcomes than particular categories of events. Using a sample of adults served by a large health maintenance organization in California, a growing body of research has established a strong dose–response relationship between the number of adverse childhood experiences and poor health outcomes in adulthood including alcohol and drug use, mental health, physical illness, and a variety of risk behaviours [6-8]. However, this sample includes individuals with private health insurance and, therefore, is not generalizable to people with histories of chronic homelessness and mental illness.

Few studies to date have looked at the accumulation of adverse childhood experiences and their effect on the adult lives of individuals who are homeless. Recently, Tsai, Edens and Rosenheck [11] examined the childhood profiles of 738 homeless adults and found three clusters: numerous childhood problems (21%); disrupted family structure (44%); and few childhood problems (35%). Participants with numerous childhood problems were significantly younger when first homeless and engaged in more severe drug use than other participants. Tam, Zlotnick and Robertson [12] examined the long-term effects of adverse childhood experiences on adult substance use, social service use, and employment among 397 homeless adults in Oakland. Adverse childhood experiences were positively correlated with consistent substance use over 15 months of follow-up as well as social service use. Further, consistent substance use was negatively associated with employment and social service use.

No studies to our knowledge have examined the cumulative impact of adverse childhood events on the adult lives of homeless people with mental illness. However, evidence suggests that adults with mental disorders report greater exposure to adverse childhood experiences compared to the general population [13] and that exposure may be related to symptom severity and course of illness [14,15]. Furthermore, studies have found that cumulative exposure to adverse childhood events was related to homelessness in the past 6 months among adults with severe mood disorders [14] and with psychotic disorders [15].

How are adverse childhood experiences linked to health risk behaviors and illness in adulthood? Research to date has focused on behaviors such as alcohol or drug use, smoking, or other behaviors that have immediate psychological or pharmacological benefit as coping strategies in the face of consistently high levels of stress [6-8]. However, few studies have examined the use of alcohol and other drugs in detail; for example, what frequency and type of substance use is associated with chains of risk? The current study further examines the relationship between adverse childhood events and a variety of adult health outcomes among a sample of homeless adults with mental illness in Vancouver, British Columbia. More specifically, we aim to explore the relationship between adverse childhood events, substance use disorders (including frequency, severity and type of substance), mental illness, duration of homelessness, and vocational functioning. In identifying early indicators for problematic substance use and/or homelessness, we are posing a larger question about how we might prevent or attenuate a myriad of negative health and social outcomes in adulthood.

## Methods

The Vancouver At Home Study is a randomized controlled trial involving homeless adults with mental illness in Vancouver, British Columbia. Study design and sample size were determined by the At Home/Chez Soi National Research Team which monitored activities at five different study sites [16]. Details related to the trial protocol such as CONSORT have been reported elsewhere [16,17]. The current study focuses on baseline data from one study site (Vancouver) prior to randomization and does not incorporate any longitudinal findings.

Eligibility criteria included legal adult status (19 years and older), current mental disorder on the MINI International Neuropsychiatric Interview (MINI) [18], and being absolutely homeless or precariously housed. Absolute homelessness was defined as living on the streets or in an emergency shelter for at least the past seven nights with little likelihood of obtaining secure accommodation in the upcoming month. Precariously housed was defined as living in a rooming house, hotel or other transitional housing; in addition, individuals must have experienced at least two episodes of absolute homelessness in the past year, or one episode lasting for at least four weeks in the past year.

Participants were recruited through referral from over 40 agencies available to homeless adults in Vancouver; the majority was recruited from homeless shelters, drop-in centres, homeless outreach teams, hospitals, community mental health teams, and criminal justice programs. We specifically targeted organizations that serve women, youth, aboriginal peoples, and gay/lesbian individuals in order to obtain as diverse and representative a sample as possible. Referral was typically initiated by service providers and a preliminary screening for eligibility (e.g., duration of homelessness, mental health and substance use problems), was conducted via telephone with the referral agent. All participants met face-to-face with a trained research interviewer who explained procedures, obtained informed consent, and confirmed study eligibility. A cash honorarium of $5 was provided to the participant for the screening process. Institutional ethics board approval was obtained through Simon Fraser University and the University of British Columbia.

If the individual met all study criteria, they were enrolled as a participant and the baseline interview commenced, consisting of a series of interviewer-administered questionnaires including socio-demographic characteristics, psychiatric symptoms, substance use, physical health, service use, and quality of life [17]. Participants received a further cash honorarium of $30 upon completion of the baseline interview which typically took 90 minutes to complete. The following analyses are based upon data from the baseline questionnaires of 497 participants recruited from October 2009 to June 2011 and data from the Adverse Childhood Experiences scale [19], which was administered 18 months after baseline.

### Variables of interest

Childhood events were assessed 18 months after the baseline interview using the Adverse Childhood Experiences (ACE) scale [19], which consists of 17 questions pertaining to age 18 or younger. The ACE includes three categories of childhood abuse: psychological abuse (2 questions), physical abuse (2 questions), contact sexual abuse (2 questions); and two categories of neglect: emotional (2 questions) and physical (2 questions). In addition, the ACE inquires about four categories of exposure to household dysfunction during childhood: parental separation or divorce (1 question), exposure to substance abuse (1 question), mental illness (1 question), violent treatment of mother or stepmother (3 questions), and incarceration (1 question) in the household. Participants received a positive score for a category if they responded “yes” to one or more of the questions in a particular category, for a maximum score of 10. Response options included Yes, No, Don’t know or Decline. Only a response of “yes” was recorded as a positive endorsement of items on the ACE. The response “don’t know” was recorded as a negative response and declining to respond was considered as missing data.

With regard to mental disorders, Severe Cluster includes at least one of current Psychosis, Mood Disorder with Psychotic Features, and Hypomanic or Manic Episode, as identified through the MINI or documented physician diagnosis. Less Severe Cluster includes at least one of current Major Depressive Episode, Panic Disorder, and Post-traumatic Stress Disorder. Suicidality, Alcohol Dependence, and Substance Dependence were also identified using the MINI. Frequency and type of substance use over the past month were recorded using the Maudsley Addiction Profile (MAP) [20]. Physical illness was assessed by self-report using a checklist of 30 chronic health conditions (lasting longer than six months). Blood-borne infectious disease consisted of positive self-report diagnosis of HIV, Hepatitis B or Hepatitis C. Vocational functioning included two variables: (1) have you ever had a job that lasted for at least one year? (yes/no) and (2) are you currently employed in paid work? (yes/no) Psychometric properties for all measures are provided in previous manuscripts [16,17].

### Statistical analyses

Comparisons of categorical data between participants who completed or did not complete the ACE were conducted using Pearson’s chi-square or Fisher’s exact test. Comparisons of numeric variables (e.g., age at enrolment) between groups were conducted using the Student t test and Wilcoxon’s rank-sum test. Univariate and multivariable logistic regression analyses were used to model the independent associations between ACE total score and a series of a priori outcome variables. Each outcome variable was modeled in both univariate and multivariate settings using ACE total score as an independent risk factor. Outcome variables that were significant at the p ≤ 0.10 level were considered for the multivariable logistic regression analyses using the same set of controlling variables (age at enrolment, gender, ethnicity, educational attainment, and level of need) chosen based on previous literature [7,8,11,12]. Both unadjusted and adjusted odds ratios and 95% confidence intervals (CI) are reported as effect sizes and all p-values are two-sided. SPSS-21 was used to conduct these analyses. Missing values ranging from zero to 2% for all outcome and controlling variables in the regression analysis were excluded.

## Results

In total, 497 participants completed the baseline questionnaire. Of the total sample, 413 participants (83%) were located for the 18 month follow-up interview and 364 of these participants (88%) provided a valid response on all ACE items. Declined items ranged from 9.2% (physical abuse) to 10.9% (maternal violence) and “don’t know” responses ranged from 2.2% (psychological and physical abuse) to 9.7% (household mental illness). Table 1 presents the baseline characteristics for the full baseline sample (n = 497) and for participants who completed the ACE (n = 364). At baseline, the majority of participants who responded to the ACE was male (71%) and White (55%); the mean age at enrollment was 41.0 (SD = 10.6) years; and the mean age when first homeless was 29.0 (SD = 13.1) years. The median duration of lifetime homelessness was 36 months (IQR: 12–84 months). Compared to the baseline sample, participants who completed the ACE were more likely to be categorized as “moderate” than “high” needs (p ≤ 0.05), based on an algorithm that considered type of mental disorder, history of psychiatric hospitalization, substance dependence and/or criminal justice involvement, and community functioning [17]. Otherwise, there were no significant differences at baseline between the full sample and participants who completed the ACE.

**Table 1:** Socio-demographic, mental disorder, and substance use-related characteristics for Vancouver At Home study participants (n = 497)

**Variable**	**Total sample (n = 497)**	**Participants with valid ACE total score (n = 364)**	**Participants with missing or declined responses (n = 133)**	**P value**
	**N (%)**	**N (%)**	**N (%)**	
Need level (High)	297 (60)	208 (57)	89 (67)	0.049*
Gender (Male)	359 (73)	255 (71)	104 (78)	0.103
Age at enrolment visit				
Youth	36 (7)	24 (7)	12 (9)	0.344
25-44 years	281 (57)	202 (56)	76 (59)	
> 44 years	180 (36)	138 (38)	42 (32)	
Ethnicity				
Aboriginal	77 (15)	62 (14)	15 (11)	0.251
White	280 (56)	199 (55)	81 (61)	
Other	140 (28)	103 (28)	37 (28)	
Incomplete high school	280 (57)	210 (58)	70 (53)	0.270
Marital status (Single)	343 (70)	250 (69)	93 (72)	0.570
Precariously housed	109 (22)	76 (21)	33 (25)	0.544
Duration of homelessness				
Lifetime (>36 months)^1^	234 (48)	173 (48)	61 (46)	0.697
Longest single period (>1 yr)^2^	245 (50)	184 (51)	61 (47)	0.373
First homeless prior to age 25 yrs	214 (44)	152 (42)	62 (47)	0.313
Overall health (Poor)	67 (13)	53 (14)	14 (10)	0.240
Type of mental disorder				
Less severe	264 (53)	202 (56)	62 (47)	0.079^+^
Severe	363 (73)	258 (71)	105 (79)	0.073^+^
Multiple (≥2) mental disorders	240 (48)	180 (49)	60 (45)	0.392
Alcohol dependence	121 (24)	91 (25)	30 (23)	0.574
Substance dependence	288 (58)	218 (60)	70 (53)	0.126
High suicidality	87 (17)	69 (19)	18 (13)	0.159
Blood-borne infectious disease	157 (32)	118 (33)	39 (30)	0.540
Multiple (≥3) physical illness	344 (69)	253 (70)	91 (68)	0.817
Age first drunk (≤13 yrs)	164 (47)	51 (40)	215 (46)	0.153
Age of first drug use (≤13 yrs)	140 (42)	43 (35)	183 (40)	0.178
Daily substance use	143 (29)	105 (29)	38 (29)	0.952
Daily drug use	93 (25)	33 (25)	126 (25)	0.867
Injection drug use	88 (18)	64 (18)	24 (19)	0.834
Poly-substance use				
Two or more	257 (52)	193 (53)	64 (49)	0.397
Three or more	148 (30)	113 (31)	35 (27)	0.345
Poly-drug use				
Two or more	188 (38)	145 (40)	43 (33)	0.150
Three or more	108 (22)	84 (23)	24 (18)	0.253
Weekly alcohol use	111 (22)	85 (23)	26 (20)	0.394
Daily alcohol use	26 (5)	20 (5)	6 (5)	0.678
Daily marijuana use	70 (14)	49 (13)	21 (16)	0.509

^1^Dichotomized based on median score (3 years).

^2^Dichotomized based on median score (1 year).

*p ≤ 0.05.

+p ≤ 0.10.

The proportion of positive responses for the ten categories included in the ACE ranged from 20% for a household member being incarcerated to 54% for psychological abuse (often experiencing an adult in the household swear, insult or humiliate the participant, or act in a way that made the participant afraid that they might be physically hurt; see Table 2). Only 12% of participants did not endorse any of the ACE items and 42% positively endorsed five or more items. The mean ACE total score was 3.9 (SD = 2.8) (see Table 2).

**Table 2:** Prevalence of adverse childhood experiences (ACE) among Vancouver At Home study participants

	**N (%)**
***Overall score (n = 364)***	
0	43 (12)
1	45 (12)
2	50 (14)
3	41 (11)
4	33 (9)
5-10	152 (42)
Mean (SD)	3.9 (2.8)
Median (range)	4 (0–10)
***Child abuse***	
Emotional abuse (n = 374)^1^	203 (54)
Physical abuse (n = 375)	186 (50)
Sexual abuse (n = 370)	104 (28)
Emotional neglect (n = 369)	168 (45)
Physical neglect (n = 371)	106 (29)
***Household dysfunction***	
Parental separation or divorce (n = 371)	197 (53)
Mother treated violently (n = 368)	90 (24)
Substance abuse (n = 373)	196 (53)
Mental illness (n = 370)	134 (36)
Incarceration (n = 370)	76 (20)

^1^Number of participants who provided a valid response to each item on the ACE.

Bivariate comparisons by ACE total score are summarized in Table 3. Participants with higher ACE scores were significantly more likely to share certain socio-demographic characteristics (i.e., Aboriginal ethnicity, incomplete high school, having children under age 18), and were significantly more likely to report a number of negative health outcomes related to physical health (i.e., blood-born infectious diseases, rating overall health as “poor”), mental health (i.e., less severe cluster of mental disorders, multiple mental disorders) and substance use (i.e., alcohol and/or substance dependence, early initiation of alcohol and/or drug use, daily alcohol and/or drug use).

**Table 3:** ACE total score by socio-demographic, physical health, mental disorder and substance use variables (n = 364)

**Variable**	**Mean (SD)**	**P value**
**Socio-demographic variables**		
Need Level		
High	3.8 (2.8)	0.423
Moderate	4.0 (2.8)	
Gender		
Male	3.7 (2.8)	0.071^+^
Female	4.3 (2.8)	
Age at enrolment		
19-24 years	3.5 (2.7)	0.672
25-44 years	4.0 (2.8)	
> 44 years	3.8 (2.8)	
Ethnicity		
Aboriginal	4.8 (3.0)	0.004**
White	3.9 (2.7)	
Other	3.4 (2.7)	
Education		
Completed high school	3.3 (2.6)	0.001***
Incomplete high school	4.3 (2.8)	
Marital status		
Single (never married)	3.8 (2.6)	0.204
Other	4.2 (3.1)	
Housing status		
Precariously housed	3.9 (2.8)	0.570
Absolutely homeless	3.7 (2.8)	
Duration of homelessness (lifetime)		
> 36 months	3.8 (2.7)	0.711
≤ 36 months	3.9 (2.8)	
Duration of homelessness (longest single episode)		
> 12 months	3.6 (2.7)	0.032*
≤ 12 months	4.2 (2.8)	
Age of first homelessness		
< 25 years	3.8 (2.8)	0.413
≥ 25 years	4.0 (2.8)	
**Physical health**		
Blood-borne infectious disease		
No	3.6 (2.7)	0.008**
Yes	4.5 (2.9)	
Multiple (≥3) physical illness		
No	3.1 (2.7)	<0.001***
Yes	4.2 (2.8)	
Overall health		
Fair/good/excellent	3.7 (2.7)	0.029*
Poor	4.6 (3.1)	
**Mental disorders (past month)**		
Less severe cluster		
No	3.4 (2.6)	0.001**
Yes	4.3 (2.9)	
Severe cluster		
No	4.1 (2.6)	0.314
Yes	3.8 (2.9)	
Multiple (≥2) mental disorders		
No	3.4 (2.6)	<0.001***
Yes	4.4 (2.9)	
Alcohol dependence		
No	3.7 (2.7)	0.005**
Yes	4.6 (2.8)	
Substance dependence		
No	3.7 (2.7)	0.005**
Yes	4.6 (2.8)	
Suicidality		
High	3.7 (2.7)	0.038*
No/low/moderate	4.5 (2.9)	
**Substance use (past month)**		
Age first drunk		
Before 14 years	4.6 (2.7)	<0.001***
14 years or after	3.3 (2.7)	
Age first used drugs		
Before 14 years	4.8 (2.7)	<0.001***
14 years or after	3.5 (2.7)	
Frequency of substance use (including alcohol)		
Less than daily/none	3.6 (2.7)	0.006**
Daily	4.5 (2.9)	
Frequency of drug use		
Less than daily/none	3.6 (2.7)	0.001***
Daily	4.7 (2.9)	
Frequency of marijuana use		
Less than daily/none	3.8 (2.7)	0.015*
Daily	4.8 (3.0)	
Frequency of alcohol use		
Less than weekly/none	3.8 (2.8)	0.089^+^
Weekly or more	4.3 (2.8)	
Injection drug use		
No	3.8 (2.8)	0.148
Yes	4.3 (2.8)	
Poly-substance use		
Two or less	3.7 (2.8)	0.056^+^
Three or more	4.3 (2.7)	
Poly-drug use		
Two or less	3.8 (2.8)	0.122
Three or more	4.3 (2.6)	

*p ≤ 0.05.

+ p ≤ 0.10.

**p≤0.01.

***p≤0.001.

Unadjusted (UOR) and adjusted odds ratios (AOR) and 95% CI for variables included in the univariate and multivariable analyses are presented in Table 4. Results from the multivariable logistic regression analyses indicate that ACE total score independently predicted meeting criteria for the less severe cluster of mental disorder(s) (AOR: 1.13), Alcohol Dependence (AOR: 1.11), Substance Dependence (AOR: 1.09), high risk of suicidality (AOR: 1.11), and two or more mental disorders (AOR: 1.15); positive self-report of infectious disease (AOR: 1.09), three or more chronic physical illnesses (AOR: 1.15), and “poor” overall health (AOR: 1.12); early initiation (prior to age 14 years) of alcohol (AOR: 1.17) and/or drugs (AOR: 1.20), current daily substance use (AOR: 1.10), daily drug use (AOR: 1.14), and daily marijuana use (AOR: 1.16). Further, a significant positive trend was observed between ACE total score and a longest single episode of homelessness of one year or more (AOR: 1.07) and past month use of three or more substances (AOR: 1.07).

**Table 4:** Logistic regression analysis for socio-demographic, mental illness, and substance use-related outcomes based on ACE total score (n = 364)

**Outcome**^**1**^	**Unadjusted OR (95% CI)**	**p value**	**Adjusted OR (95% CI)**^**2**^	**p value**
**Duration of homelessness**				
Cumulative lifetime (>3 years)	1.10 (0.94, 1.09)	0.710		
Longest single episode (>1 year)	1.09 (1.01, 1.17)	0.032*	1.07 (0.99, 1.16)	0.108+
Age first homeless (<25 years)	1.03 (0.96, 1.11)	0.412		
**Mental disorder**				
Less severe cluster	1.14 (1.05, 1.23)	0.001**	1.13 (1.04, 1.23)	0.004**
Severe cluster	0.96 (0.88, 1.04)	0.313		
Alcohol dependence	1.13 (1.04, 1.23)	0.006**	1.11 (1.01, 1.21)	0.030*
Substance dependence	1.11 (1.02, 1.19)	0.012*	1.09 (1.00, 1.19)	0.040*
High suicidality	1.10 (1.01, 1.21)	0.039*	1.11 (1.01, 1.23)	0.032*
Multiple (≥2) mental disorders	1.15 (1.07, 1.24)	<0.001***	1.15 (1.06, 1.24)	0.001**
**Physical health**				
Blood-borne infectious diseases (HIV/HCV/HBV)	1.11 (1.03, 1.21)	0.008**	1.09 (1.01, 1.19)	0.039*
Multiple (≥3) physical illness	1.17 (1.07, 1.27)	0.001**	1.15 (1.05, 1.26)	0.015*
Overall health (poor)	1.12 (1.01, 1.25)	0.030*	1.12 (1.00, 1.25)	0.047*
**Substance use**				
Age first drunk (<14 years)	1.19 (1.10, 1.29)	<0.001***	1.17 (1.08, 1.28)	<0.001***
Age of first drug use (<14 years)	1.20 (1.10, 1.30)	<0.001***	1.20 (1.10, 1.31)	<0.001***
IV drug use	1.07 (0.98, 1.18)	0.149		
Daily substance use	1.12 (1.03, 1.22)	0.007**	1.10 (1.01, 1.20)	0.027*
Daily illicit drug use	1.15 (1.06, 1.25)	0.001**	1.14 (1.04, 1.25)	0.005**
Daily hard drug use (no marijuana)	1.09 (0.98, 1.20)	0.104+	1.05 (0.95, 1.17)	0.349
Weekly alcohol use	1.08 (0.99, 1.18)	0.089+	1.06 (0.97, 1.16)	0.191
Daily marijuana use	1.14 (1.02, 1.27)	0.017*	1.16 (1.04, 1.31)	0.010*
Poly-substance (≥3) use	1.08 (1.00, 1.17)	0.057+	1.07 (0.99, 1.17)	0.099+
Poly-drug (≥3) use	1.07 (0.98, 1.17)	0.123		
Poly-drug (≥2) use	1.05 (0.97, 1.13)	0.257		

*p ≤ 0.05 **p ≤ 0.01 ***p ≤ 0.001 + p ≤ 0.10.

^1^Separate binary logistic regression analyses (univariate and multivariable) were conducted for each outcome using ACE total score (continuous measure) as an independent variable.

^2^Each multivariable model was controlled for age (continuous), gender (male vs. female), ethnicity (Aboriginal, Caucasian, Other), need level (High vs. Moderate), and education (completed vs. incomplete high school).

## Discussion

Among our sample of homeless adults with mental illness, we found a strong relationship between the breadth of exposure to abuse or household dysfunction during childhood (ACE total score) and a number of indicators of poor mental and physical health as well as problematic substance use in adulthood. ACE total score independently predicted meeting criteria for a current mental disorder in the less severe cluster (i.e., major depressive episode, panic disorder or post-traumatic stress disorder), multiple mental disorders, and high risk of suicide; infectious disease, three or more chronic physical conditions, and poor self-rated health; alcohol and/or substance dependence, early initiation of alcohol and/or drug use, and daily use of any substance, illicit drugs, and marijuana. These findings suggest that the impact of adverse childhood experiences on adult health and social functioning is strong and cumulative among homeless individuals with mental illness.

Of concern was the very high rate of adverse events reported by our sample: 65% reported personally experiencing abuse, 53% reported experiencing neglect, and 79% reported household dysfunction. The mean number of adverse childhood experiences reported was 4. Only 24% of participants reported 1 or zero adverse childhood experiences, 34% reported 2 to 4 events, and 42% reported 5 to 10 events. Rates of adverse childhood experiences in our study were two to nine times higher than those reported by Dube et al. [7] using a large HMO sample. Our findings are similar to those reported by Wu et al. [21] who administered the Life Stressor Checklist-Revised to adults with concurrent mental illness and substance dependence in a residential drug treatment program: 16% of participants reported 1 or zero adverse childhood experiences, 49% reported 2 to 4 events, and 34% reported 5 or more events. Sullivan et al. [2] reported that about one-quarter of their sample of homeless adults with mental illness experienced residential instability as children and over one-third witnessed violence in the home or personally experienced abuse. These authors concluded that homeless people with mental illness appear to receive a “double dose” of disadvantage in the form of poverty as well as family instability and violence. Our findings suggest that childhood adversity among homeless adults with mental illness is much more pervasive and cumulative, and likely contributes to a number of chronic health problems in adulthood.

Consistent with other studies, multiple adverse childhood experiences predicted a variety of adult health problems including physical illness [22,23], mental illness [24] and substance use problems [12,25,26]. As expected, ACE score is predictive of depressive and anxiety disorders, including post-traumatic stress disorder, rather than disorders that are typically characterized as “severe” such as psychotic and bipolar disorders. However, the relationship between ACE score and physical illness and substance abuse suggests a complex syndrome that can be very severe in terms of its impact and duration. ACE total score independently predicted a range of substance use problems in our adult sample, including early initiation of drug and/or alcohol use (before age 14). Along with other studies, our findings suggest that daily drug use is a common mediator for a range of early risk factors [27]. Thus, it appears that abuse of alcohol and other drugs places an individual at greater risk of homelessness, but is not a direct causal factor [28]. Previous research using our sample of homeless adults with mental disorders found that daily drug use significantly predicts the duration of homelessness [29] as well as the severity of mental health symptoms [30].

Cross-sectional, retrospective data cannot disentangle the unique predictors of homelessness and mental illness, but it is likely that negative childhood experiences have both direct and indirect effects on participants’ history of homelessness. Documentation of these underlying common factors points to a broad range of vulnerabilities for homelessness and mental illness. These common factors increase the complexity of personal problems as well as the duration of homelessness [29]. Therefore, substance dependence, especially when concurrent with mental illness among homeless populations, is not only a clinical problem but also a critical indicator for a range of other social and psychological problems that may need to be addressed before homelessness can be resolved.

According to Briere’s [31] self-trauma model, beyond its initial negative effects, early and cumulative childhood trauma interrupts normal child development, conditions negative affect to abuse-related stimuli, and interferes with the usual acquisition of self-capacities such as affect regulation skills. Reduced affect regulation places an individual at risk for being more easily overwhelmed by emotional distress associated with memories of trauma, and increases the likelihood of using dissociation and other avoidant coping strategies in adolescence and adulthood. In this way, impaired affect regulation leads to reliance on avoidance strategies which, in turn, further prevent the development of self-regulation capacities. This negative cycle is exacerbated by the individual’s tendency to repetitively re-experience cognitive-emotional memories of the traumatic event in an effort to process conditioned emotional responses and distorted cognitive schema -- a process that can further overwhelm self-regulation and produce distress. Therefore, in addressing the long-term impact of adverse childhood experiences, the role of family context and environment (e.g., parenting and attachment) must be considered alongside avoidance strategies such as substance use.

### Implications

Children who have experienced trauma are more likely to experience trauma and abuse in the future [32]. Furthermore, victims of childhood trauma often engage in post-victimization behavior in the form of violence against self or others and poor personal and occupational functioning [33]. The experience of homelessness increases the likelihood that an individual will witness or experience trauma, and homelessness itself is considered as a traumatic experience that interrupts routines and damages social networks [34]. Among homeless populations, having a mental illness and bearing witness to multiple violent events are predictive of increased severity of trauma symptoms [35], placing the individual at higher risk for social and functional difficulties including reduced social support and impaired work performance [12].

Research on early indicators of risk for homelessness has important implications for the prevention of homelessness as well as intervention and service provision. Given the high prevalence and long-term negative consequences associated with adverse childhood experiences (in general as well as homeless populations), increased attention to primary, secondary and tertiary prevention strategies is needed. Primary prevention of adverse events will ultimately require societal changes that improve the quality of family and household environments during childhood, particularly for poor households. Longitudinal evaluations of early intervention programs (secondary prevention) such as Head Start [36] and the Nurse Family Partnership [37] have documented the prevention of a range of health, social and justice related problems with vulnerable groups (e.g., low income children and first-time mothers).

Prevention also requires increased recognition of the effects of childhood trauma as well as a better understanding of the behavioral coping strategies that are commonly adopted to reduce the emotional impact of these experiences. However, psychological assessment and treatment for children and adolescents is often grossly inadequate. Where psychosocial interventions are available, improved coordination between mental health professionals, general practitioners, child protection and public health workers, and families is greatly needed in order to better understand how social, emotional, and medical problems are linked throughout the lifespan.

### Limitations

A potential weakness of studies with retrospective reporting of childhood experiences is recall bias. Longitudinal follow-up of adults whose childhood abuse was documented has shown that their retrospective reports of such abuse are likely to underestimate actual occurrence [38]. Therefore, difficulty recalling childhood events likely results in misclassification (classifying people who truly were exposed to ACEs as unexposed) that would bias our results toward the null hypothesis. Also, substance use is likely under-reported by participants particularly given that the baseline questionnaires were administered prior to randomization to supported housing or usual care. In addition, the number of participants who declined to respond to items on the ACE was relatively high, and suggests an attempt to avoid thinking about distressing past events. If this is the case, it would result in further under-reporting of adverse events in our sample. Other than level of need, we found no significant differences between participants who completed vs. those who did not complete the ACE. Finally, there may be mediators of the relationship between childhood experiences and adult health status other than the risk factors we examined such as childhood conduct problems or foster care placement.

The retrospective and cross-sectional nature of our data preclude the kind of modeling required to identify the primary adult outcomes related to adverse childhood events. Further longitudinal research is required to more fully understand the developmental and social sequelae related to childhood adverse events. Further research is also needed to understand how social factors regulate behaviours or distribute individuals into risk groups and how those social factors push individual trajectories towards or way from adverse outcomes.

## Conclusions

Our research, along with others’, shows that the problems experienced by the majority of homeless adults with mental illness have longstanding histories dating back to childhood. Poverty, family instability, damaging psychological experiences, and general household distress are all disproportionately present in the childhood backgrounds of our participants. These early experiences likely work both directly and indirectly to produce risk for homelessness in various ways, shaping, influencing and constraining the intra- and interpersonal resources that children can draw from adults [4].

## Competing interests

This research was funded by Health Canada and the Mental Health Commission of Canada. The views expressed herein solely represent the authors. The authors declare no competing interests.

## Authors’ contributions

MLP drafted the manuscript and oversaw data collection; AM conducted the statistical analyses; JMS contributed to study design and critical editing of the manuscript. All authors reviewed the final draft. All authors read and approved the final manuscript.

## Pre-publication history

The pre-publication history for this paper can be accessed here:

http://www.biomedcentral.com/1471-2458/14/350/prepub
